# Comprehensive DNA methylation analysis of tissue of origin of plasma cell-free DNA by methylated CpG tandem amplification and sequencing (MCTA-Seq)

**DOI:** 10.1186/s13148-019-0689-y

**Published:** 2019-06-24

**Authors:** Xiaomeng Liu, Jie Ren, Nan Luo, Huahu Guo, Yuxuan Zheng, Jingyi Li, Fuchou Tang, Lu Wen, Jirun Peng

**Affiliations:** 1 Beijing Advanced Innovation Center for Genomics (ICG), College of Life Sciences, Peking University, Beijing, 100871 China; 2 Department of Surgery, Beijing Shijitan Hospital, Capital Medical University, Beijing, 100038 China; 3 Biomedical Pioneering Innovation Center (BIOPIC), College of Life Sciences, Peking University, Beijing, 100871 China; 4 Department of Surgery, Beijing Shijitan Hospital, Peking University Ninth School of Clinical Medicine, Beijing, 100038 China; 5 Peking-Tsinghua Center for Life Sciences, Peking University, Beijing, 100871 China; 6 Sichuan Cancer Hospital & Institute, Sichuan Cancer Center, School of Medicine, University of Electronic Science and Technology of China, Chengdu, 610041 China

**Keywords:** Circulating cell-free DNA, DNA methylation, Next-generation sequencing

## Abstract

**Background:**

Comprehensive analysis of the tissue of origin of plasma cell-free DNA (cfDNA) remains insufficient. A genome-scale DNA methylation method for this analysis is of both biological and clinical interest.

**Methods:**

We used the methylated CpG tandem amplification and sequencing (MCTA-Seq), which is a genome-scale DNA methylation method, for analyzing cfDNA. We performed MCTA-Seq to pair plasma cfDNA and white blood cell genomic DNA from 14 healthy individuals for comparative analysis, with eight tissues being analyzed for identifying tissue-specific markers. The relative contributions of multiple tissues to cfDNA were calculated for plasma cfDNA obtained from healthy adults (*n* = 25), cholelithiasis patients (*n* = 13), liver cirrhosis patients (*n* = 17), hepatocellular carcinoma patients (*n* = 30), and acute pancreatitis patients (*n* = 8).

**Results:**

We identified a total of 146 tissue-specific hypermethylation markers. Simulation analysis showed that MCTA-Seq can accurately measure DNA fractions contributed by multiple tissues to cfDNA. We demonstrated that the liver is the major non-hematopoietic tissue contributing to plasma cfDNA in healthy adults. The method also detected increases in the liver-derived DNA in the blood from patients with liver diseases, which correlate with an increase in the liver enzyme level. Furthermore, the results indicated that blood cells make a major contribution to the elevation of cfDNA levels in acute pancreatitis, liver cirrhosis, and hepatocellular carcinoma patients. Finally, we characterized a novel set of tissue-specific hypermethylation markers for cfDNA detection, which are located within the intragenic regions of tissue-specific highly expressed genes.

**Conclusions:**

We have used MCTA-Seq for simultaneously measuring cfDNA fractions contributed by multiple tissues. Applying this approach to healthy adults and liver and pancreas disease patients revealed the tissue of origin of cfDNA. The approach and the identified markers should facilitate assessing the cfDNA dynamics in a variety of human diseases.

**Electronic supplementary material:**

The online version of this article (10.1186/s13148-019-0689-y) contains supplementary material, which is available to authorized users.

## Background

The biological and diagnostic applications of circulating cell-free DNA (cfDNA) have attracted great interest in recent years. The non-invasive prenatal testing of fetal chromosomal aneuploidy by cfDNA sequencing has been widely used in the clinic [1, 2]. Also, “liquid biopsy” of tumor-specific mutations in cfDNA has great promise for cancer diagnosis and monitoring [3].

While genetic-based approaches have been successfully used, DNA methylation has long been studied as a promising epigenetic cfDNA biomarker because of its stable and informative nature [4, 5]. A unique characteristic of DNA methylation vs genetic-based markers is tissue specificity [6–10]. Different cell types have distinct DNA methylation patterns, which can be used to distinguish the tissue of origin of cfDNA. Indeed, individual tissue-specific DNA methylation markers have recently been shown to be sensitive for blood-based detection of tissue cell death in several diseases [11–15]. Genome-wide and targeted approaches have also recently been used to inspect the fractions of cfDNA contributed by multiple tissues [16–19].

Despite these progresses, DNA methylation analysis of the tissue of origin of plasma cfDNA is still insufficient. For example, a number of previous studies have shown that cfDNA levels are elevated in many clinical disorders including cancer [20, 21], autoimmune diseases [22], liver diseases [23–26], intensive exercise [27], and acute medical emergencies such as acute pancreatitis [28], trauma [29], stroke [30], myocardial infarction [31], and sepsis [32]. The cellular origin of the increased cfDNA level remains incompletely clarified. Additionally, complementary approaches are needed for identifying novel tissue-specific methylation markers for cfDNA detection.

We recently developed methylated CpG tandem amplification and sequencing (MCTA-Seq) for cfDNA analysis [33]. As a genome-scale DNA methylation method that enriches methylated CpG islands, MCTA-Seq is informative, sensitive, and cost-effective and is suitable for efficient screening of novel cfDNA methylation markers. Here, we extended this method to assess the relative contributions of multiple non-hematopoietic tissues to plasma cfDNA. We have analyzed 60 tissue samples and 85 plasma samples, identifying a total of 146 tissue-specific methylation markers and investigating the tissue of origin of plasma cfDNA in healthy individuals and liver and pancreas disease patients.

## Methods

### Subject

All subjects were recruited from the Department of Surgery, Beijing Shijitan Hospital, Capital Medical University of China (which is also the Ninth School of Clinical Medicine, Peking University). The study was approved by the Ethics Committee of Beijing Shijitan Hospital, Capital Medical University. Written informed consents were obtained from all subjects before inclusion in the study.

### DNA extraction and MCTA-Seq library preparation

Commercialized genomic DNAs for normal tissues, including the lung (*n* = 2), stomach (*n* = 2), colon (*n* = 2), kidney (*n* = 2), pancreas (*n* = 2), muscle (*n* = 2), and skin (*n* = 2) were purchased from BioChain. The DNAs were extracted from white blood cells (WBC) using the DNeasy Blood & Tissue Kit (Qiagen) according to the manufacturer’s protocol. Plasma cfDNA was obtained as described previously. The cfDNA concentration was quantified using the Qubit dsDNA HS Kit (Invitrogen, Q32854). The MCTA-Seq library was prepared and sequenced as described previously with small modifications [33]. Briefly, cfDNA obtained from 2 mL plasma (up to 24 ng) or 400 ng tissue gDNA were treated by bisulfite and purified using the MethyCode bisulfite conversion kit (Invitrogen). All bisulfite-converted cfDNA or 60 ng bisulfite-converted gDNA (quantified using the Qubit ssDNA Kit, Q10212) were then amplified using MCTA-Seq primers A and B as described previously. The full amplicon was amplified in a 50-μL reaction by adding a 30-μL solution containing 1× Ex Taq Buffer, 250 μM each dNTP, 2 μM primer C (5′-AATGATACGGCGACCACCGAGATCTACACTCTTTCCCTACACGACGCTCTTCCGATCT-3′) and 2 μM primer D (5′-CAAGCAGAAGACGGCATACGAGATCTGATCGTGACTGGAGTTCAGACGTGTGCT-3′), and the reaction was subjected to 14 cycles of 95 °C for 30 s, 65 °C for 30 s, 72 °C for 1 min, and a final cycle of 72 °C for 5 min. Then, instead of processing individual samples, we pooled six samples with different Illumina index sequences by taking 30-μL reactions for each sample and then purified. The pooled product was resolved on a 3% agarose gel (Takara, Agarose LM SIEVE, D614), and the fraction between 180 and 250 bp was excised and then purified. For the tissue sample, the product is usually ready for serving as the library for sequencing. For the plasma sample, one to two additional rounds of amplification (using primers QP1 (5′-AATGATACGGCGACCACCGA-3′) and QP2 (5′-CAAGCAGAAGACGGCATACGA-3′)) and gel purification are usually needed to clean up the primer dimers and obtain enough materials for sequencing.

### Data processing

FASTQ format R2 reads were first processed and filtered as described previously (step i to step v), using hg19 as the reference sequence [33]. Instead of considering all alleles within a CGI as the unit for calculation as in our previous study, we focused on the fully methylated molecules (FMMs) amplified from a CGCGCGG as the unit for calculation in the present study. Specifically, for a certain CpG site (from the 1st to the 10th) downstream a CGCGCGG, the methylation value is calculated as the number of FMMs, i.e., all CpGs between this CpG site and the CGCGCGG site were methylated, normalized by the total number of reads uniquely mapped to the whole human genome, and expressed as methylated alleles per million mapped reads (MePM). We calculated the MePM value for both the plasma and tissue samples.

### Identification of tissue-specific methylation markers

Tissue-specific CGCGCGG methylation markers among 8 normal tissue types, including the liver, lung, stomach, colon, kidney, pancreas, muscle, and skin, were identified using the following criteria: (i) a tissue-specific index (*τ*) was defined [34], and a threshold *τ* > 0.9 was used to define tissue-specific methylation; (ii) MePM > 10 in the most hypermethylated tissue; (iii) 90% percentile MePM = 0 in a training set of 29 WBC samples; (iv) average MePM < 1 in the liver if the most hypermethylated tissue is not the liver; and (v) the distance between the CpG site and the CGCGCGG site is less than 60 bp. These criteria yielded 59, 9, 20, 13, 18, and 8 markers for the liver, the stomach, the colon, the kidney, the pancreas, and the skin, respectively. Since no markers for the lung and muscle were identified using these criteria, we omitted the tissue-specific index restriction and identified 8 lung markers and 11 muscle markers.

### Deconvolution analysis for MCTA-Seq cfDNA tissue mapping

The mathematical relationship between the methylation values (MePM) of plasma and the corresponding MePM in each tissue of marker *i* can be expressed by the following formula:

$$ {\overline{\mathrm{MP}}}_{\mathrm{i}}={\sum}_k\kern0.5em {\overline{MT}}_{ik}\ast {P}_k, $$

in which $\overline{\mathrm{MP}}$_*i*_ represents the methylation value of the tissue-specific CGCGCGG marker *i* in plasma cfDNA; $\overline{\mathrm{MT}}$_ik_ represents the methylation value of marker *i* in tissue *k*; and *P*_*k*_ represents the proportional contribution of tissue *k* to plasma cfDNA. The particle swarm optimization program [35, 36] was used to solve the simultaneous equations. In practice, we added up the markers except the maximum of each tissue type as one tissue-specific methylation marker. We also omitted the contribution of WBC since the markers were selected as having very low methylation values in WBC (90 percentile MePM = 0). Thus, a total of 8 simultaneous equations representing 8 non-hematopoietic tissue types were generated to be solved. The median value of ten runs was used as the readout. The range of *P*_*k*_ should fulfill the expectation of 0 to 1. To further eliminate any effect from non-specific methylation in WBC, the average tissue fraction values in fourteen paired WBC samples (0.015%, 0, 0.36%, 0, 0, 0, 0.066%, and 0 for the liver, lung, stomach, colon, kidney, pancreas, muscle, and skin, respectively) were subtracted from the measured tissue fractions. In addition, the measured tissue fractions less than the average values plus three standard deviations of WBC samples (0.11%, 0, 2.0%, 0.082%, 0.036%, 0.023%, 0.57%, and 0 for the liver, lung, stomach, colon, kidney, pancreas, muscle, and skin, respectively) were set to zero. Combining the sensitivities calculated in the simulation analysis, we estimate that the low detection limits of the MCTA-Seq cfDNA tissue mapping method are 0.25% for the liver and pancreas; 0.5% for the colon, kidney, and skin; and 2% for the lung, stomach, and muscle.

For the simulation analysis, MCTA-Seq data of the non-hematopoietic tissue types and WBC (from 14 healthy individuals) were mixed in serial ratios with tissue fractions ranging from 0.25 to 16%, in a total of approximately 3 million uniquely mapping paired-end reads. We then plotted the expected and measured tissue fractions.

For analyzing the plasma of HCC patients, we only used 85 markers that are not hypermethylated in HCC tissues (liver-specific markers: liver tumor tissue vs the adjacent non-cancerous liver tissues, one-tailed MWW test, *P* > 0.05; other tissue specific markers: 90 percentile of liver tumor tissues < 10) to exclude the effects of the cancer-released DNA on the tissue mapping.

### The relationship between the tissue-specific hypermethylation markers and the tissue-specific genes

RNA-Seq data of normal human tissues were downloaded from the Human Protein Atlas. A threshold *z*-score > 1.5 was used to define tissue-specific genes. The intragenic region was defined as starting at 300-bp downstream of a RefGene transcription start site (TSS) and ending at 300-bp downstream of a RefGene transcription end site.

### Bioinformatics and statistical analysis

Custom R scripts and R packages were used to construct boxplots, heatmaps, and bar plots and to perform statistical analysis. The MePM of plasma and paired WBC from normal individuals were normalized to the same duplication rate (plasma, 1.4 ± 0.3; WBC, 1.3 ± 0.1 (mean ± SD)).

## Results

### Genome-wide DNA methylation comparison between plasma cfDNA and WBC gDNA

**Fig. 1:**
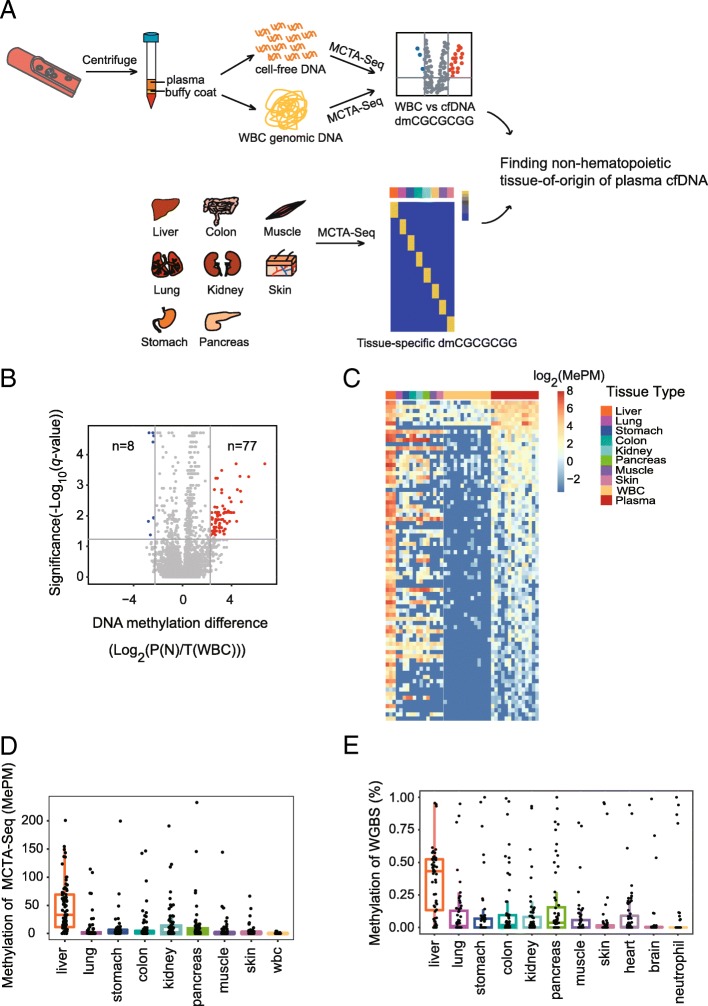
Genome-wide DNA methylation comparison between plasma and WBC. **a** Schematic diagram of the strategy for a systematic and unbiased examination of the non-hematopoietic tissue of origin of plasma cfDNA. **b** Volcano plot showing differentially methylated CGCGCGGs (dmCGCGCGGs) between plasma and WBC. The *x*-axis shows the fold change of the average methylation value between plasma cfDNA and WBC gDNA, and the *y*-axis shows the *q* value as the FDR analog of the *P* value (−Log_10_(*q* value)) for a two-tailed MWW test of differences between two groups. The horizontal gray line indicates statistical significance (FDR < 0.05). **c** A heatmap showing methylation of the dmCGCGCGGs in different tissues and 14 paired WBC and plasma samples. **d**, **e** Boxplots of the dmCGCGCGGs showing **d** the methylation values (methylated alleles per million mapped reads, MePM) of MCTA-Seq or **e** the methylation levels in the published DNA methylome data

We designed a systematic and unbiased strategy to investigate the non-hematopoietic tissue of origin of plasma cfDNA in healthy individuals. This strategy first identified the regions that are significantly hypermethylated in plasma cfDNA vs white blood cell (WBC) genomic DNA (gDNA) extracted from matched buffy coats using the MCTA-Seq method. Then, the tissue-specific methylation signatures of these regions were resolved to elucidate which non-hematopoietic tissues contribute to plasma cfDNA (Fig. 1a).

We performed MCTA-Seq on matched plasma and WBC samples obtained from 14 healthy individuals. The plasma cfDNA data were reported in our previous study [33]. A schematic diagram of the sample collection is shown in Additional file 1: Figure S1. The WBC samples were sequenced to a median depth of 6 million raw reads pair similar to the plasma samples (see Additional file 2: Table S1 for sequencing information). A total of 77 CGCGCGGs were found to be significantly differentially methylated (dmCGCGCGGs) in the plasma cfDNA (average methylation (alleles per million mapped reads, MePM) fold changes of cfDNA vs WBC > 5, false discovery rate (FDR) < 0.05, two-tailed Mann-Whitney-Wilcoxon (MWW) test, Fig. 1b). In contrast, only 8 dmCGCGCGGs were identified in WBC gDNA.

To obtain the tissue-specific methylation pattern, we performed MCTA-Seq on seven major tissues, including the lung (*n* = 2), stomach (*n* = 2), colon (*n* = 2), kidney (*n* = 2), pancreas (*n* = 2), muscle (*n* = 2), and skin (*n* = 2). The data of the normal liver tissues (*n* = 3) were reported in our previous study [33]. Notably, we found that most plasma dmCGCGCGGs were hypermethylated in the liver (Fig. 1c). The methylation values of these dmCGCGCGGs in the liver were substantially higher than those in other tissues (MePM: median 33.2 in the liver vs 0.4 in the lung, 2.1 in the stomach, 0.6 in the colon, 1.0 in the kidney, 1.1 in the pancreas, 0 in the muscle, 0.4 in the skin, and 0.1 in WBC, *P* < 0.01, two-tailed MWW test, Fig. 1d). We also validated the methylation values of these regions by using published DNA methylome data [8, 37, 38]. The results showed that these dmCGCGCGGs in cfDNA have a median methylation level of 42% in the liver, which is considerably higher than that in the seven other tissues (*P* < 0.01, two-tailed MWW test, Fig. 1e). In addition, these dmCGCGCGGs have low methylation levels in other tissues, including brain and heart (0% in the brain, 0% in the heart).

### Identification of tissue-specific methylation markers

To verify the results and to determine whether any other tissues also contribute detectably to cfDNA, we used a complementary approach that first identified the tissue-specific methylation CGCGCGG markers and then examined whether they are differentially hypermethylated in cfDNA. MCTA-Seq was performed on an additional set of 29 WBC samples as a training group for selecting markers with very low methylation levels in WBC (90th percentile = 0, MePM).

**Fig. 2:**
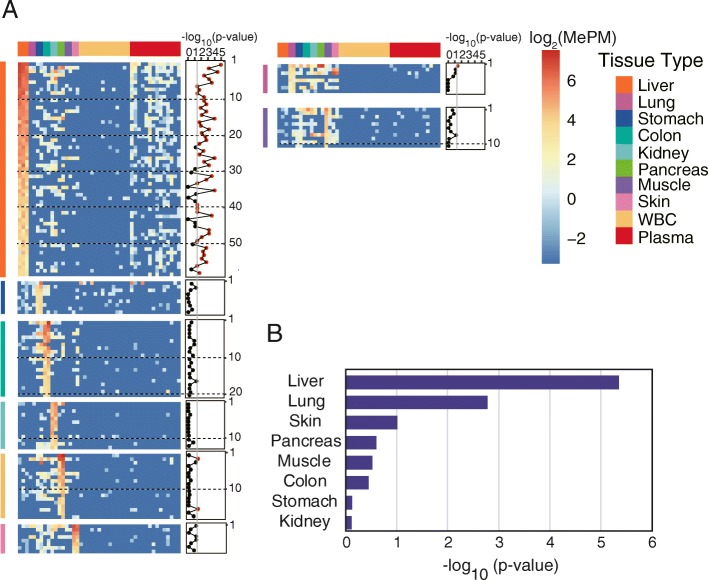
Analysis of tissue-specific methylation markers. **a** A heatmap showing methylation of the tissue-specific methylated CGCGCGGs in different tissues and 14 paired WBC and plasma samples. For each CGCGCGG, the *P* values (−Log_10_(*P* value)) for a two-tailed MWW test of differences between the WBC and plasma samples are shown. **b** Bar graphs of the *P* values (−Log_10_(*P* value)) of each set of tissue-specific methylation markers after adding up their methylation values for a two-tailed MWW test of differences between the WBC and plasma samples

We identified a total of 146 tissue-specific methylated CGCGCGGs, among which 59, 9, 20, 13, 18, 8, 8, and 11 markers are for the liver, the stomach, the colon, the kidney, the pancreas, the skin, the lung, and the muscle, respectively (see the “Methods” section). Detailed information and data of these markers are provided in Additional file 2: Tables S2 and S3. Genomic views of representative markers validated by published DNA methylome data were shown in Additional file 1: Figure S2 [8, 37, 38]. We then compared the methylation values of these markers between 14 pairs of plasma and WBC samples. To exclude the effects of sequencing depth, we also normalized the data of each pair of matched cfDNA and WBC gDNA to the same depth (Additional file 2: Table S1). The results showed that most liver-specific markers (80%, 47 out of 59) were significantly differentially methylated in cfDNA (*P* < 0.01, two-tailed MWW test, Fig. 2a), confirming that the liver prominently releases DNA into the plasma under physiological conditions. None of the other tissues showed clear evidence of release based on the analysis of individual CGCGCGG markers.

To maximize the detection sensitivity, we then combined the CGCGCGG makers for each tissue by adding up their methylation values. As expected, combining the liver markers resulted in more significant hypermethylation differences in the cfDNA vs WBC (*P* = 4.5e−6, two-tailed MWW test, Fig. 2b). Interestingly, we found that combining 8 lung markers revealed significantly differential hypermethylation in plasma cfDNA vs WBC (*P* = 1.7e−3, two-tailed MWW test, Fig. 2b). The difference was still significant when combining any 7 of 8 markers, indicating that it was not due to any individual marker (*P* < 0.01). In contrast, no significant release was found for any other tissues (*P* > 0.1).

Together, the systematic comparison and the tissue-specific marker analysis indicated that the liver is the major examined non-hematopoietic tissue that contributes to plasma cfDNA in healthy adults.

### Deconvolution analysis for plasma of healthy individuals

**Fig. 3:**
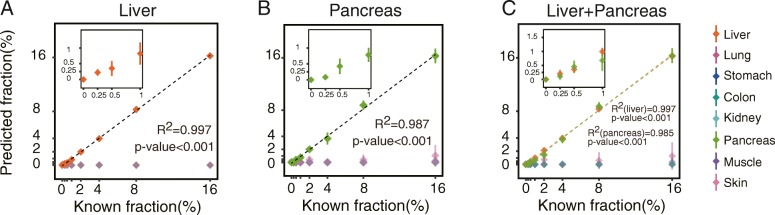
Detection sensitivity of the MCTA-Seq deconvolution analysis. The DNA percentages of different tissues estimated using the MCTA-Seq deconvolution analysis were plotted against the varying percentages of **a** the liver, **b** pancreas, and **c** the simulated mixture of the liver and pancreas in WBC. A linear fit was observed. The error bars represent means ± SD

Next, we sought to quantify the non-hematopoietic tissue DNA fractions in plasma cfDNA using deconvolution analysis. All tissue-specific CGCGCGG markers except for one stomach marker, which showed high methylation levels in 14 WBC samples, were used to set up the deconvolution algorithm (see the “Methods” section). To test the accuracy and sensitivity of the approach, we performed simulation analysis to generate a series of synthetic datasets with the tissue fraction ranging from 0.25 to 16%. The results showed linear regression lines between the simulated fractions and the estimated fractions with *R*^2^ greater than 0.95 for most tissue types (Fig. 3 and Additional file 1: Figure S3). The sensitivities, which were determined by the lowest distinguishable tissue fractions from WBC, were 0.25% for the liver and pancreas; 0.5% for the colon, kidney, and skin; and 2% for the lung, stomach, and muscle (*P* < 0.05, two-tailed *t* test, see the “Methods” section). Analyzing the synthetic data generated by mixing two tissue types, e.g., the liver and the pancreas, into WBC showed that DNA fractions of each tissue type were accurately estimated (Fig. 3).

**Fig. 4:**
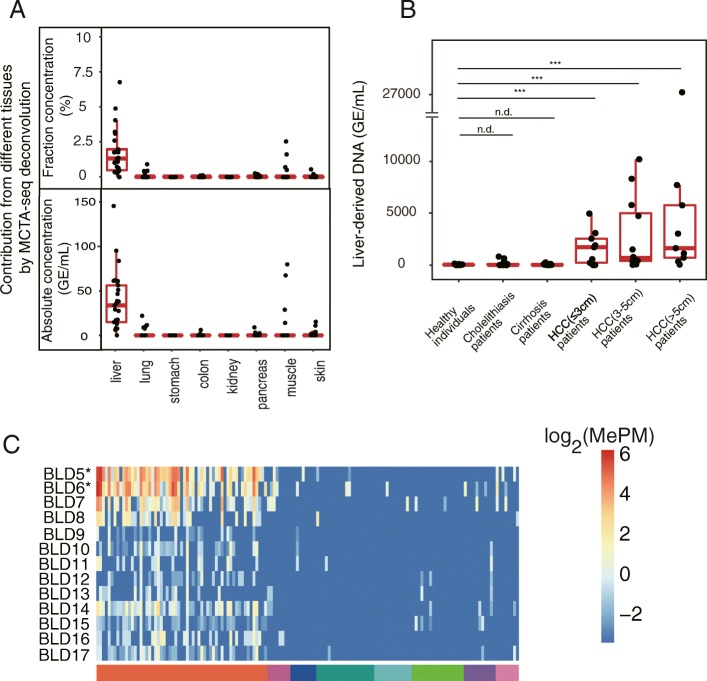
MCTA-Seq deconvolution analysis of healthy individuals and liver disease patients. **a** Boxplots showing absolute (upper panel) or fractional (lower panel) DNA fractions derived from different tissues in healthy individuals. **b** Boxplots showing absolute liver DNA fractions in the plasma of healthy individuals and liver disease patients. ***P < 0.01; nd, no difference. **c** A heatmap showing the methylation of tissue-specific CGCGCGG markers in plasma samples obtained from the cholelithiasis patients. The asterisks indicate two hepatolithiasis patients

We next calculated the percentage of DNA derived from the liver and other tissues in the plasma cfDNA of healthy individuals. We analyzed all 25 plasma samples obtained from healthy adults with measured cfDNA concentrations in our previous study [33]. The deconvolution analysis showed that the liver contributed a median fraction value of 1.3% (interquartile range (IQR) 0.48–2.0%) of plasma cfDNA (Fig. 4a and Additional file 2: Table S4). All other tissues yielded a median fraction of 0. We further calculated the absolution copies of the tissue DNAs and determined that the median absolute copy of liver-derived DNA is in these healthy individuals was 34 haploid genomic equivalents (GE) per milliliter plasma (IQR, 15–5; Fig. 4a, Additional file 2: Table S5). The liver-derived DNA fraction was not related to gender or age (Additional file 1: Figure S4).

### Deconvolution analysis for plasma from patients of liver diseases

Then, we examined plasma cfDNA obtained from the liver disease patients including cholelithiasis patients (*n* = 13), cirrhosis patients (*n* = 17), and hepatocellular carcinoma (HCC, *n* = 30) (Additional file 2: Table S6). The cholelithiasis patients include those suffering from cholecystolithiasis (*n* = 8), choledocholithiasis (*n* = 3), and hepatolithiasis (*n* = 2) (Additional file 2: Table S7). The data of the cirrhosis and HCC patients were reported in our previous study [33]. The MCTA-Seq results showed that the liver-derived DNA substantially increased in HCC patients in comparison with healthy individuals (median 26.8, 27.0, 1724.6, 692.0, 1639.7 GE/mL for the cholelithiasis patients, liver cirrhosis patients, and the HCC patients with small, middle, and large tumors, respectively, vs 33.7 GE/mL for healthy individuals, two-tailed MWW test, Fig. 4b). Notably, two hepatolithiasis patients (BLD5 and BLD6), who also showed evidence of liver damage with elevation of the liver enzyme alanine aminotransferase (ALT; normal range 9–50 U/L; 154 and 110 U/L for BLD5 and BLD6, respectively, Additional file 2: Table S7), displayed prominently higher levels of liver-derived DNA (817 and 649 GE/mL, or 30% and 25% for BLD5 and BLD6, respectively) comparing with patients suffering cholecystolithiasis and choledocholithiasis patients. The methylation values of the liver-specific markers uniformly increased in these patients, indicating authentic signals (Fig. 4c).

**Fig. 5:**
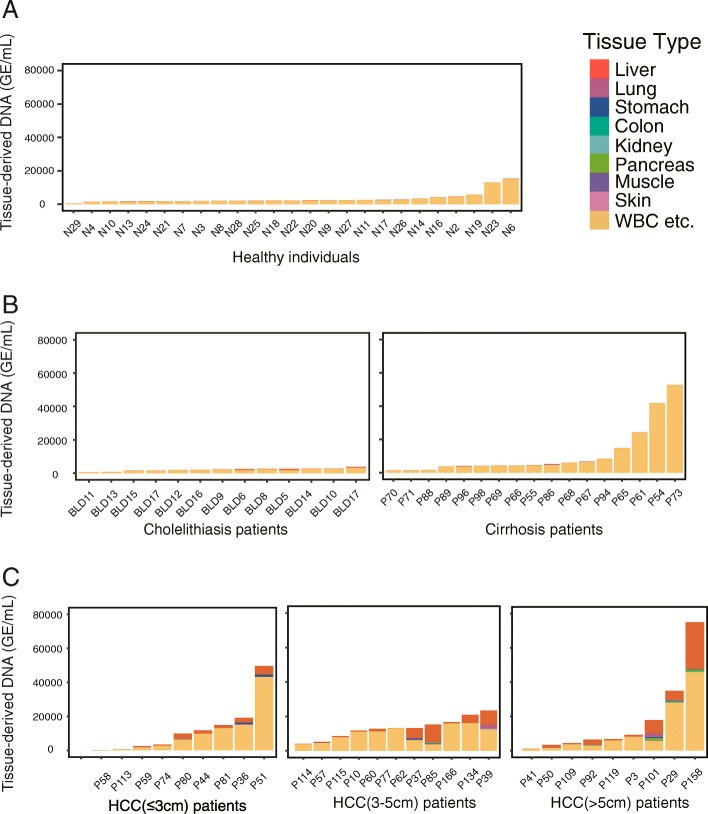
Contribution of different tissues to plasma cfDNA in **a** healthy individuals and **b** cholelithiasis, liver cirrhosis, and **c** HCC patients

Consistent with previous reports, we found that the total cfDNA concentrations were significantly higher in both liver cirrhosis and HCC patients in comparison with healthy individuals (median 7.2, 13.3, 29.9, 39.0, 20.1 ng/mL for the cholelithiasis patients, the liver cirrhosis patients, and the HCC patients with small, middle, and large tumors, respectively, vs 6.5 ng/mL for healthy individuals, two-tailed MWW test, Additional file 1: Figure S5A and Additional file 2: Table S5) [24–26]. We performed a deconvolution analysis to investigate the tissue of origin of the elevated cfDNA. The results interestingly indicated that the blood cells were the major contributor to the cfDNA increase of both the liver cirrhosis and HCC patients (Fig. 5). For the cirrhosis patients, we consistently did not detect an increase of the liver-derived DNA (Fig. 4b). For the HCC patients, though both the absolute and fractional liver-derived DNA prominently increased, the blood cells still made a major contribution to the elevated cfDNA level in most cases (Fig. 5).

### Deconvolution analysis for plasma from acute pancreatitis patients

**Fig. 6:**
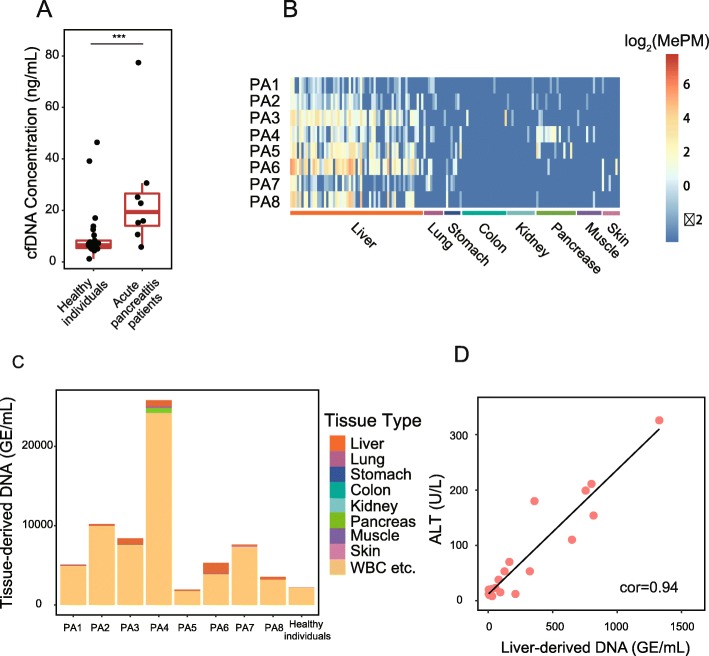
MCTA-Seq deconvolution analysis of acute pancreatitis patients. **a** Boxplots showing cfDNA concentrations of acute pancreatitis patients. Blue arrows indicate two mild cases of acute pancreatitis patients. **b** A heatmap showing the methylation of tissue-specific CGCGCGG markers in plasma samples obtained from acute pancreatitis patients. **c** Contribution of different tissues to plasma cfDNA in AP patients. **d** Correlation between absolute liver-derived DNA and ALT in cholelithiasis and AP patients

Elevated total cfDNA concentrations in acute pancreatitis (AP) patients have been reported to correlate with disease severity [23, 28]. However, the tissue of origin of the increased cfDNA is unknown. We therefore performed MCTA-Seq on plasma cfDNA obtained from eight AP patients, with six patients suffering from severe AP and two patients with mild AP (Additional file 2: Tables S6 and S8). Consistent with previous reports, we found that total cfDNA concentration was notably higher in the AP patients in comparison with the healthy individual (AP vs healthy individual: median 15 vs 6.5 ng/mL, *P* < 0.01, two-tailed MWW tests; Fig. 6a). Those patients with severe AP had a prominently higher cfDNA level than the patients with mild AP (PA5 and PA8; Fig. 6a, c; Additional file 2: Table S8), which is consistent with the previous report [28]. Unexpectedly, we only detected a clear increase in pancreas-derived DNA in patient PA4 representing 652 GE/mL or 2.8% cfDNA (Fig. 5b). The deconvolution analysis indicated that the blood cells made a major contribution to the elevated cfDNA levels (Fig. 6c). We also detected the increase in the liver-derived DNA in acute biliary pancreatitis patients (Additional file 2: Tables S5 and S8). The results of all pancreatitis and cholelithiasis patients showed that the absolute but not fractional liver-derived DNAs correlated well with ALT levels, suggesting that the absolute value is a better indication of the liver cell death than the fractional value due to the extra release from the blood cell (Spearman correlation 0.94 vs 0.58; Fig. 5d and Additional file 1: Figure S5B).

Together, we have detected liver- or pancreas-derived DNA in the plasma from patients of corresponding diseases. Our results indicated that the blood cells made a major contribution to the elevated cfDNA levels in the liver cirrhosis, HCC, and AP patients.

### A novel set of tissue-specific hypermethylation markers for cfDNA detection

**Fig. 7:**
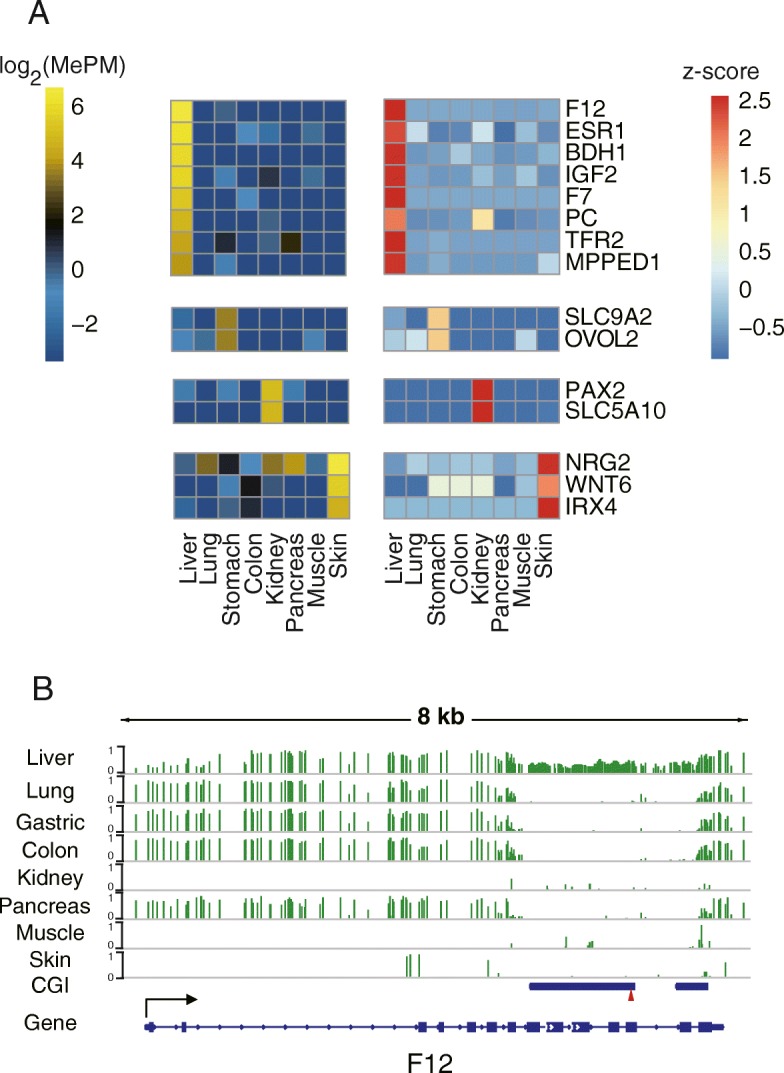
Tissue-specific hypermethylated cfDNA markers in the intragenic regions of tissue-specific genes. **a** A heatmap showing the methylation value of the gene expression of 16 tissue-specific CGCGCGG markers located in tissue-specific genes. **b** Genomic view of the F12 gene region. The red triangle indicates the CGCGCGG sequence

Recent studies have identified tissue-specific hypomethylation cfDNA markers at the promoter regions of tissue-specific genes [13, 39, 40]. Interestingly, we found that 15 of 146 tissue-specific hypermethylation markers were located within tissue-specific highly expressed genes and showed a positive correlation between the methylation value and gene expression (Fig. 7a). These genes included many typical cell type-specific genes such as the hepatocyte-specific F7, F12, and TFR2 genes, which encode the coagulation factor VII, coagulation factor XII, and transferrin receptor 2, respectively. Instead of being located at the promoter region as the hypomethylation markers, most of these hypermethylation markers were intragenic CpG islands. Genomic views of these markers validated using published DNA methylome data were shown in Fig. 7b and Additional file 1: Figure S6. Hence, our results indicated that the intragenic CGIs of tissue-specific genes can be candidate target regions for identifying tissue-specific hypermethylation markers suitable for cfDNA detection.

## Discussion

In the present study, we have used MCTA-Seq to establish a deconvolution algorithm for analyzing the tissue of origin of plasma cfDNA from healthy individuals and patients of liver and pancreatic diseases. We have also identified novel tissue-specific methylation markers useful for cfDNA detection.

For healthy individuals, our results showed that, among all eight examined non-hematopoietic tissues, the liver is the major one contributing to plasma cfDNA corresponding to a median percentage of 1.3% or 33 GE/mL in healthy adults; in contrast, other tissues give minor contributions. The result is consistent with recent studies using individual liver-specific DNA methylation markers and the DNA methylation array [14, 15, 19]. During the preparation of this manuscript, Moss et al. reported the use of an array-based method for comprehensive analysis of the tissue of origin of cfDNA [19]. Their results interestingly indicated that vascular endothelial cells and hepatocytes are the two major non-hematopoietic sources of plasma cfDNA from healthy individuals. The MCTA-Seq should be performed to vascular endothelial cells in the future.

These findings clearly demonstrate that the liver releases a substantial amount of DNA into the blood in healthy adults. This reflects the fact that the liver is the largest organ in the body, with abundant blood circulation. These results suggest a daily turnover of hepatocytes in healthy individuals [14]. Assuming that the volume of distribution of DNA is 60–70 mL/kg, the concentration of cfDNA is 6 ng/mL and corresponds to a genomic DNA content of 1000 diploid cells per milliliter, with a half-life of approximately 1 h [41–43]. The contribution of 1~2% of total plasma cfDNA of a 70-kg person means that approximately 1.2 × 10^6^ hepatocytes undergo apoptosis or necrosis with the DNA releasing to the blood per day, accounting for 0.0005% of all ~ 2.4 × 10^11^ hepatocytes in the liver [44]. This number is also largely consistent with the estimation that approximately 7.5 × 10^6^ liver stem cells divide per day [45]. Even though organs such as the colon and skin have high daily cellular turnover, we and others did not detect their DNA in the plasma, given the fact that dead cells in the mucosa of the intestinal tract and in the epidermis of the skin generally fall off of the body. We also detected potential lung-derived cfDNA in healthy individuals. However, since our method has relatively low sensitivity for detecting lung-derived DNA, we are not able to draw a conclusion in the present study.

We have detected increases in liver or pancreas-derived DNA in patients of corresponding diseases, which revealed potential clinical applications of the method. The correlation between the levels of liver-derived DNA and the liver enzyme ALT is consistent with the report of Moss et al. [19]. Comparing with the liver enzymes, the cfDNA-based assays may be useful for clinical detection of the liver damage with its distinct characteristic including a definite indication of hepatocyte death and quick clearance.

Our results interestingly indicated that the blood cells make a major contribution to the elevated cfDNA levels in the liver cirrhosis, HCC, and AP patients. Recently, by using an array-based DNA methylation deconvolution method, Moss et al. showed that the elevated cfDNA level in transplantation and sepsis patients was mostly derived from the blood cells [19]. By studying the sex-mismatched hematopoietic stem cell transplantation and liver transplantation patients, Suzan et al. also showed that exercise-induced increases in plasma cfDNA mainly originate from cells of the hematopoietic lineage [46]. These results indicate that the blood cell is an important source of increased cfDNA levels in human diseases. There should be different causes for different diseases. While strong immune reactions may lead to the increase of leukocyte-derived cfDNA in the AP patients, destruction of blood cells by hypersplenism is likely the cause of elevated hematopoietic-derived cfDNA the liver cirrhosis patients [47]. Cancer-related immune response and hypersplenism may jointly result in the increase of hematopoietic-derived cfDNA in the HCC patients, as most HCCs develop in cirrhotic livers [48].

Finally, we have identified 146 tissue-specific DNA methylation markers suitable for cfDNA detection. Since MCTA-Seq detected plenty of loci that are included in the commonly used DNA methylation array, it serves as a method for efficiently screening novel cfDNA methylation markers. Recent studies have demonstrated the utility of individual tissue-specific hypomethylation markers for detecting tissue cell death using cfDNA [12, 13, 15]. Typical tissue-specific hypomethylation markers are present in the promoter regions of highly expressed tissue-specific genes, which are specifically unmethylated in the corresponding tissue, e.g., the insulin gene promoter, which is unmethylated in insulin-producing pancreatic β cells [12]. In the present study, we identify a group of tissue-specific DNA hypermethylation markers that are suitable for cfDNA detection. In contrast to hypomethylation markers, these hypermethylation markers are located within the intragenic CpG islands of highly expressed cell type-specific genes, such as the hepatocyte-specific F7, F12, and TFR2 genes. Combined with the information of gene expression, these loci are more explicit cell type-specific markers. The tissue-specific DNA methylation pattern of intragenic CGIs has been previously reported, and a mechanism of transcription-mediated DNA methylation has been shown [49, 50]. The work done by ourselves and others provides a rational basis for identifying new individual DNA methylation markers for the non-invasive detection of tissue-specific cell death.

## Conclusions

We have developed a MCTA-Seq deconvolution approach for simultaneously assessing the proportions of plasma cfDNA derived from multiple non-hematopoietic tissues. Applying this approach to healthy individuals and liver and pancreas disease patients revealed tissue of origin of plasma cfDNA. We have also identified novel tissue-specific cfDNA hypermethylation markers. The approach and the identified markers have many research and diagnostics applications in a broad spectrum of human diseases.

## Additional files

Additional file 1: Genome-scale DNA methylation analysis of tissue of origin of plasma cell-free DNA. **Figure S1.** Schematic diagram of the study design and sample collection. **Figure S2.** Genomic views of representative tissue-specific methylation markers. The red triangle indicates the CGCGCGG sequence. **Figure S3.** Detection sensitivity of the MCTA-Seq deconvolution analysis. The DNA percentages of different tissues estimated using the MCTA-Seq deconvolution analysis were plotted against the varying percentages of A) the lung, stomach, colon, kidney, muscle, and skin. A linear fit was observed. The error bars represent means ± SD. **Figure S4.** The relationship between liver-derived DNA fraction and A) gender or B) age. **Figure S5**. MCTA-Seq deconvolution analysis of healthy individuals, liver disease patients, and acute pancreatitis patients. A) Boxplots showing cfDNA concentration (ng/mL) in the plasma of healthy individuals and liver disease patients. ***P < 0.01; *P < 0.1; nd, no difference. B) Correlation between liver-derived DNA fraction concentration and ALT in cholelithiasis and AP patients. **Figure S6.** Genomic view of the A) F7, BDH1, TFR2, MPPED1, IGF2, ESR1, and PC gene regions. The red triangle indicates the CGCGCGG sequence. (PDF 2827 kb)

Additional file 2: Supplementary **Tables S1–S8.** (XLSX 180 kb)

## Data Availability

All MCTA-Seq data were deposited to the NCBI under accession number GSE118690, go to https://www.ncbi.nlm.nih.gov/geo/query/acc.cgi.
