# Supplementary figures and images for: Comprehensive DNA methylation analysis of tissue of origin of plasma cell-free DNA by methylated CpG tandem amplification and sequencing (MCTA-Seq)
Source: Clin Epigenetics. 2019 Jun 24;11:93. doi: 10.1186/s13148-019-0689-y (PMC6591962; doi:10.1186/s13148-019-0689-y)

Figure S1

A

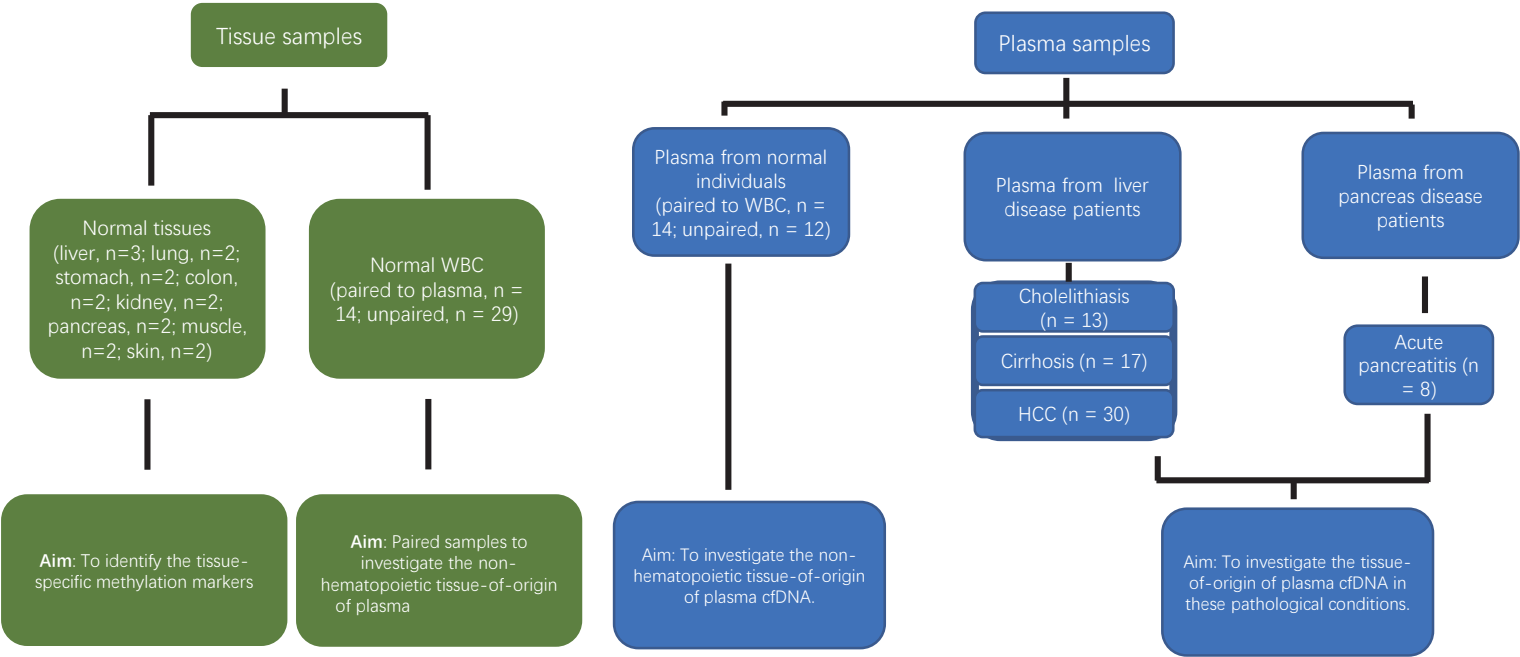

Figure S2

A

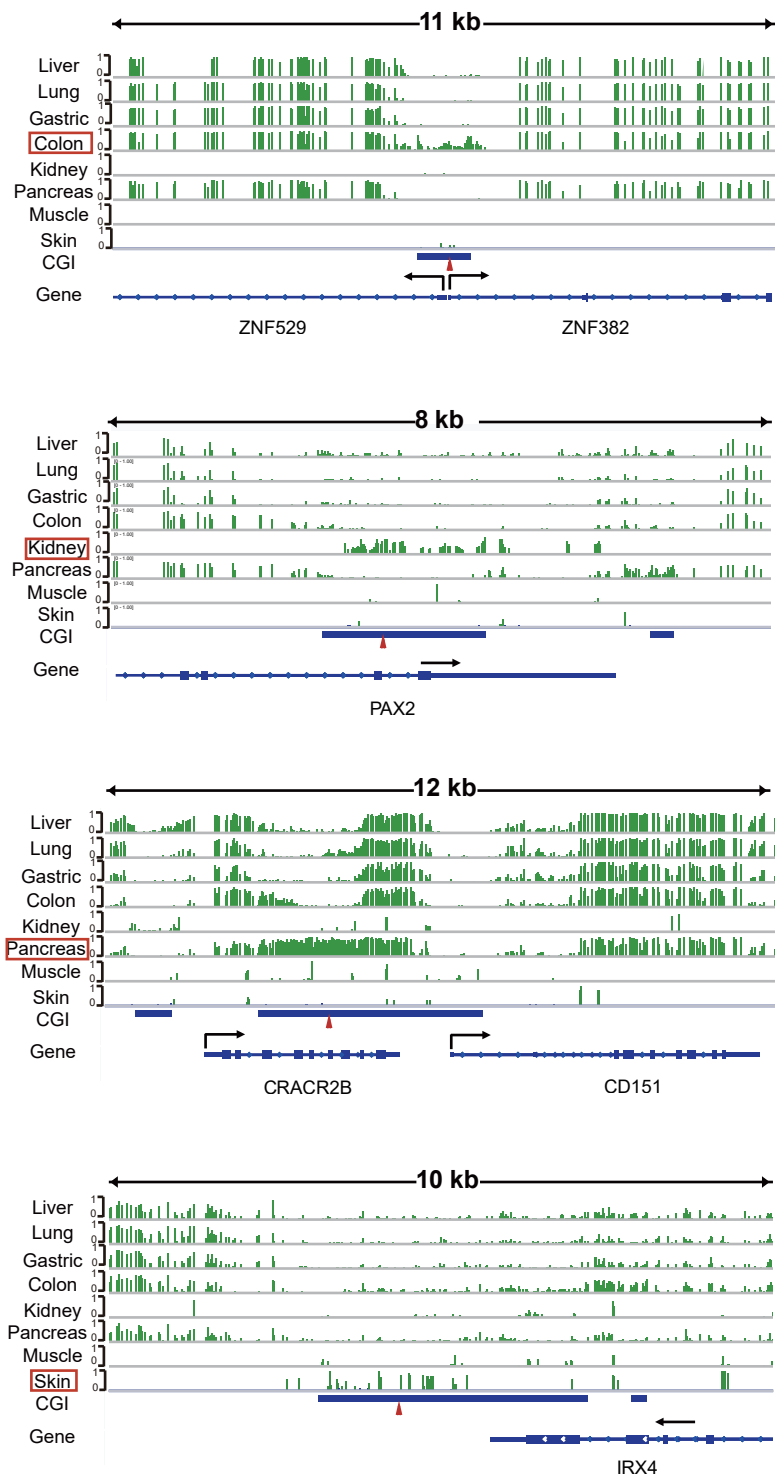

Figure S3

A

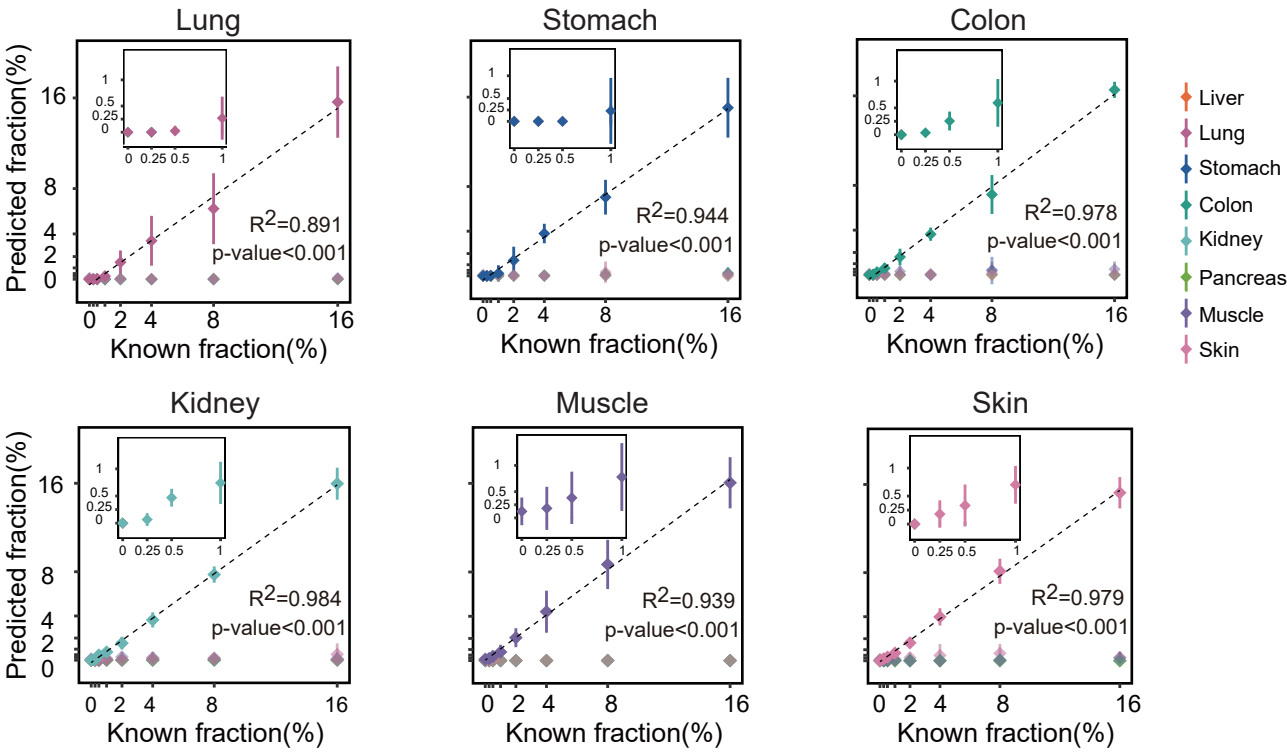

Figure S4

A

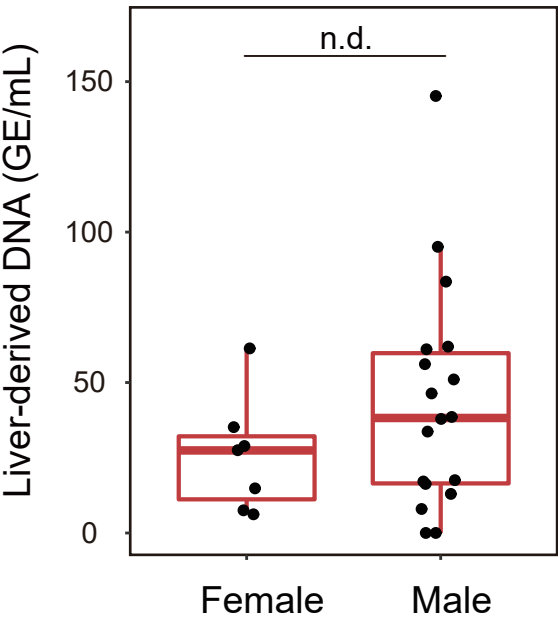

B

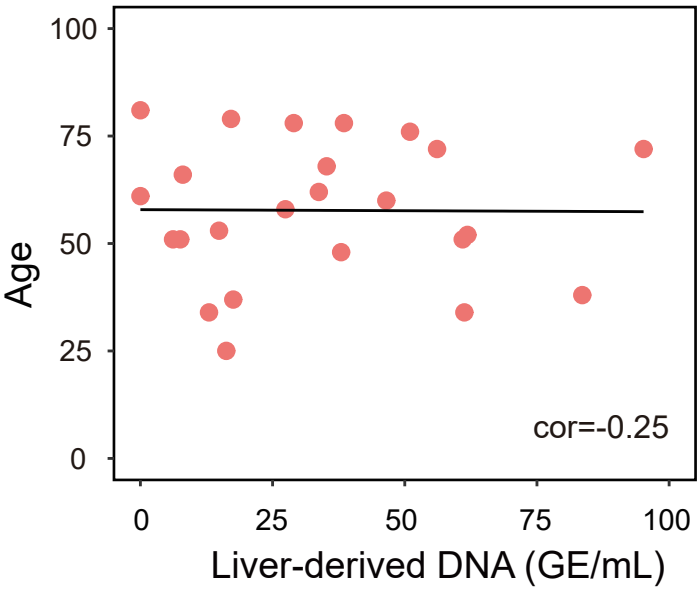

Figure S5

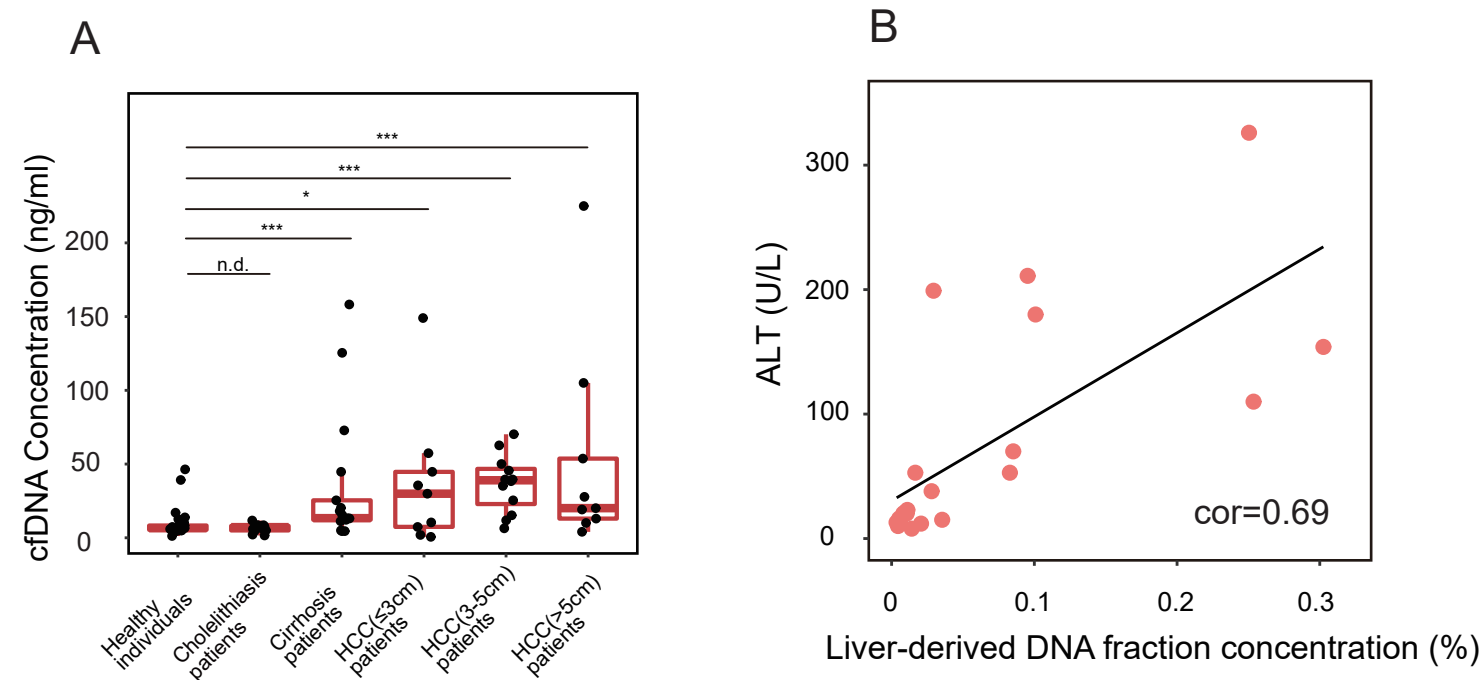

Figure S6

A

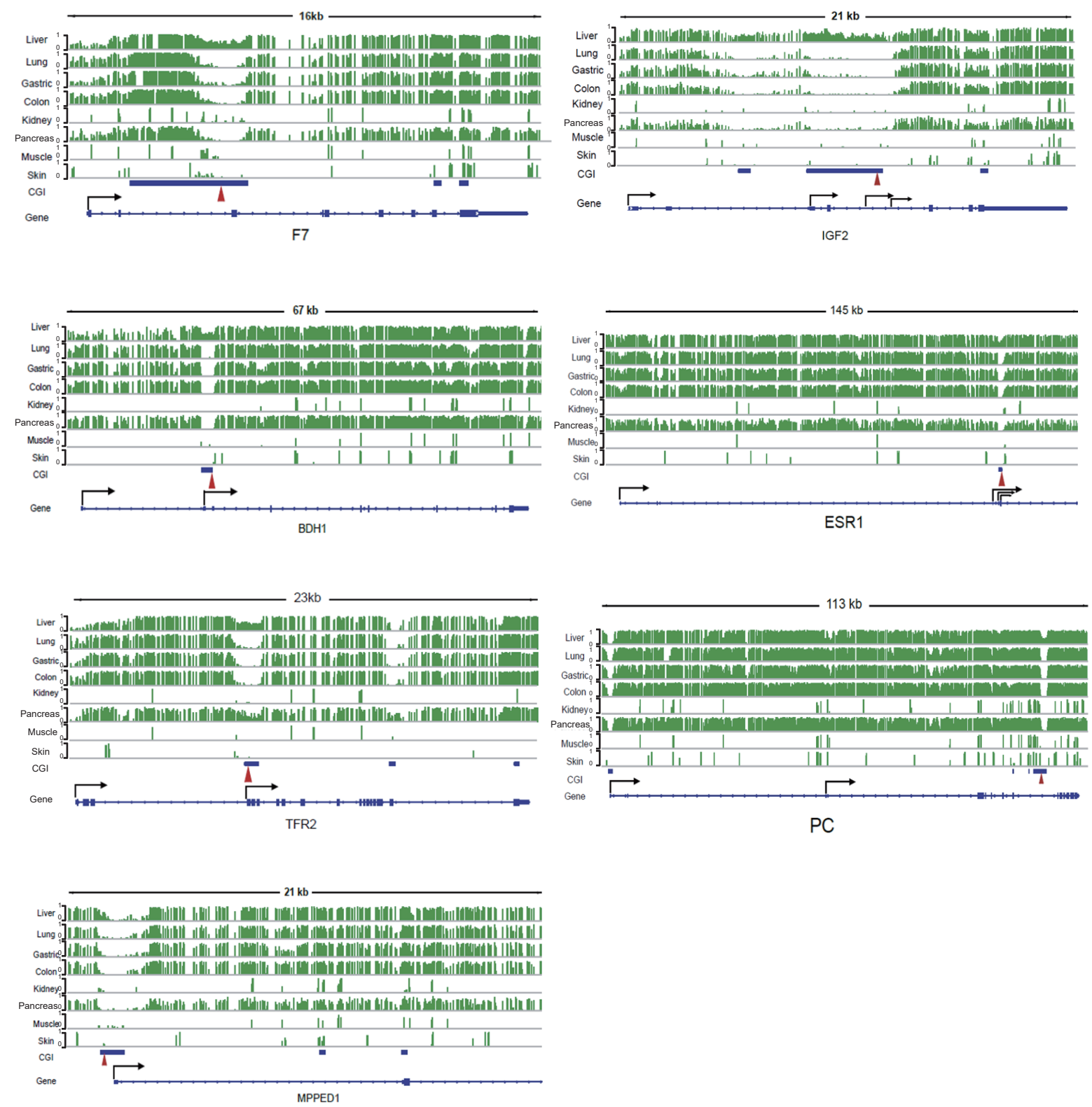

Supplement: Supplementary file 1 — Genome-scale DNA methylation analysis of tissue of origin of plasma cell-free DNA. Figure S1. Schematic diagram of the study design and sample collection. Figure S2. Genomic views of representative tissue-specific methylation markers. The red triangle indicates the CGCGCGG sequence. Figure S3. Detection sensitivity of the MCTA-Seq deconvolution analysis. The DNA percentages of different tissues estimated using the MCTA-Seq deconvolution analysis were plotted against the varying percentages of A) the lung, stomach, colon, kidney, muscle, and skin. A linear fit was observed. The error bars represent means ± SD. Figure S4. The relationship between liver-derived DNA fraction and A) gender or B) age. Figure S5. MCTA-Seq deconvolution analysis of healthy individuals, liver disease patients, and acute pancreatitis patients. A) Boxplots showing cfDNA concentration (ng/mL) in the plasma of healthy individuals and liver disease patients. ***P < 0.01; *P < 0.1; nd, no difference. B) Correlation between liver-derived DNA fraction concentration and ALT in cholelithiasis and AP patients. Figure S6. Genomic view of the A) F7, BDH1, TFR2, MPPED1, IGF2, ESR1, and PC gene regions. The red triangle indicates the CGCGCGG sequence. (PDF 2827 kb) [file 13148_2019_689_MOESM1_ESM.pdf]
